# RVG-functionalized reduction sensitive micelles for the effective accumulation of doxorubicin in brain

**DOI:** 10.1186/s12951-021-00997-z

**Published:** 2021-08-21

**Authors:** Jiangkang Xu, Xiaoye Yang, Jianbo Ji, Yuan Gao, Na Qiu, Yanwei Xi, Anchang Liu, Guangxi Zhai

**Affiliations:** 1 Department of Pharmaceutics, Key Laboratory of Chemical Biology (Ministry of Education), School of Pharmaceutical Sciences, Shandong University, Jinan, 250012 People’s Republic of China; 2 Department of Pharmacy, Qilu Hospital of Shandong University, 107 WenhuaXilu, Jinan, 250012 China; 3 Department of Clinical Pharmacy, School of Pharmaceutical Sciences, Shandong University, 44 WenhuaXilu, Jinan, 250012 China

**Keywords:** Cell-penetrating peptide, RVG, Glioma, Brain targeting, Drug accumulation

## Abstract

**Background:**

Glioblastoma is a lethal neoplasm with few effective therapy options. As a mainstay in the current treatment of glioma at present, chemotherapeutic agents usually show inadequate therapeutic efficiency due to their low blood brain barrier traversal and brain targeting, together with tumor multidrug resistance. Novel treatment strategies are thus urgently needed to improve chemotherapy outcomes.

**Results:**

Here, we report that nanomedicines developed by functionalizing the neurotropic rabies virus-derived polypeptide, RVG, and loading reduction-sensitive nanomicelles (polymer and doxorubicin) enable a highly specific and efficacious drug accumulation in the brain. Interestingly, curcumin serves as the hydrophobic core of the polymer, while suppressing the major efflux proteins in doxorubicin-resistant glioma cells. Studies on doxorubicin-resistant rat glioma cells demonstrate that the RVG-modified micelles exhibit superior cell entry and antitumor activity. In vivo research further showed that RVG modified nanomicelles significantly enhanced brain accumulation and tumor inhibition rate in mice, leading to a higher survival rate with negligible systemic toxicity. Moreover, effective suppression of recurrence and pulmonary metastatic nodules were also determined after the RVG-modified nanomicelles treatment.

**Conclusions:**

The potential of RVG-modified nanomicelles for glioma was demonstrated. Brain accumulation was markedly enhanced after intravenous administration. This unique drug delivery nanoplatform to the brain provides a novel and powerful therapeutic strategy for the treatment of central nervous system disorders including glioma.

**Graphic abstract:**

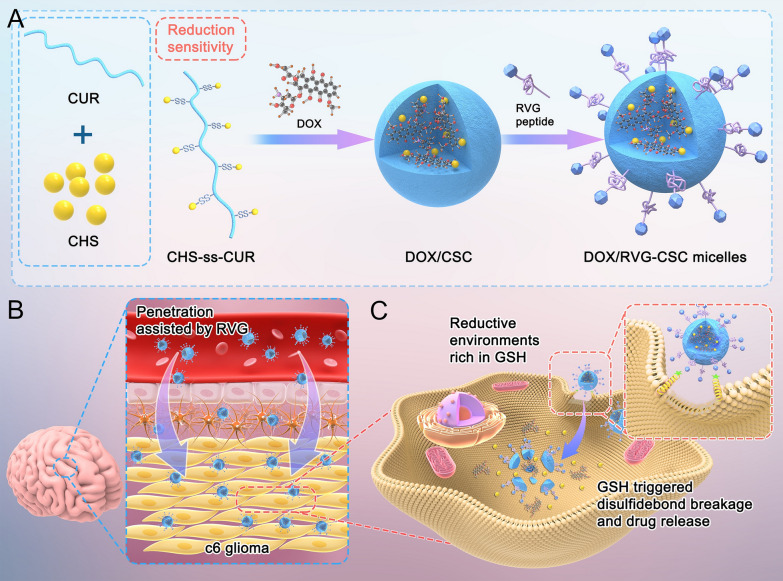

**Supplementary Information:**

The online version contains supplementary material available at 10.1186/s12951-021-00997-z.

## Introduction

As the most frequent of all aggressive cerebral malignancies (6 cases per 100 thousand individuals) [1, 2], glioblastoma multiforme (GBM) has no effective therapeutic options, still causing at least 10,000 deaths each year for the past 30 years [3]. Surgical resections usually result in a poor prognosis because of strong metastatic and invasiveness [4]. The median survival time upon diagnosis exists no more than 15 months, even though there is a combination of traditional treatments (including chemoradiotherapy and radiotherapy) [5–7]. Residual tumor cells interweave with the surrounding brain tissue and are protected by the blood–brain barrier (BBB), a bidirectional biological membrane that separates the systemic circulation and the brain parenchyma. Cerebrovascular endothelial cells and astrocytes are tightly stacked to form this dense physical barrier. Moreover, tens of thousands of different efflux pumps and selective transporters on the surface of various cells also constitute a biochemical barrier [5, 8]. The expression of the energy-dependent efflux pump, P-glycoprotein (P-gp), in GBM and the cerebral vascular endothelial lumen allows the rapid clearance of chemotherapeutic agents that are not blocked by the BBB [9]. Poor brain-targeted efficiency and multidrug resistance (MDR) cause tumor cells to exhibit an extremely low response to monotherapy even with recurrence from the resection margin cavity [10].

The emergence of nanomaterials seems to hold promise for improving the current situation over the inefficiencies and severe side effects of previous GBM chemotherapy [11, 12]. Various strategies have been pursued in the last decade to achieve enhanced BBB penetration and effective intracephalic drug release. Considerable efforts have been made, such as the opening of tight junctions, tight junction relaxation, carrier-mediated transport, and dual targeting ligand modification, to endow the formulation with the ability to target diseased tissue [13]. Some functionalized nanocarriers serve as intelligent delivery systems profiting from their environmental responsiveness, controllable particle size, and optimized surface properties to provide an effective way to treat brain diseases including GBM [14–16]. Although these intelligent nanodelivery platform-based chemotherapies overcome certain bottlenecks to some extent, combinational regimens need to break all cerebral therapeutic constraints: natural biological membranes, low brain-targeted efficiency, and MDR, in conclusion, poorly effective accumulation of chemotherapy drugs in the brain. The development of effective targeted therapeutic agents for GBM remains an urgent problem demanding prompt solution in cancer therapy.

**Fig. 1:**
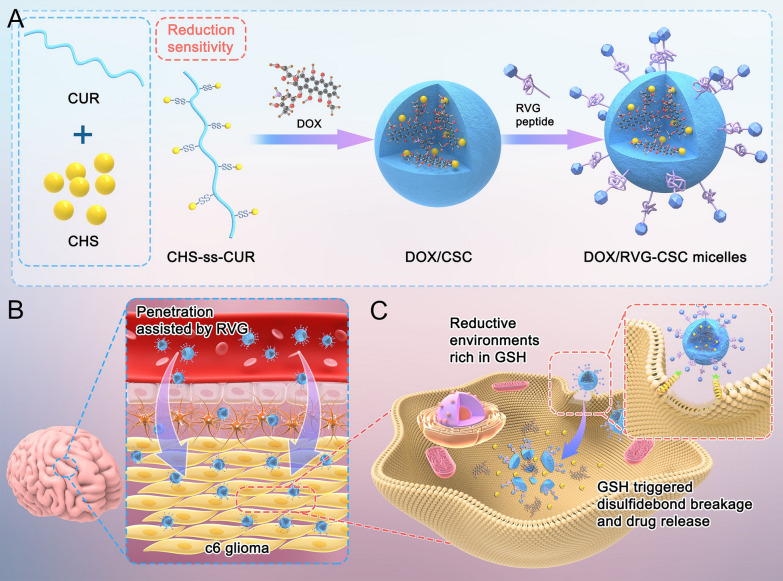
Penetrated BBB and achieved efficient drug delivery to mice glioblastoma by an RVG peptide-directed, doxorubicin-loaded and curcumin-assisted reduction sensitivity nanomicelle (DOX/RVG-CSC). **A** Formation schematic diagram of DOX/RVG-CSC. **B** Process by which DOX/RVG-CSC penetrates the blood–brain barrier and accumulates in brain. **C** Active tumor cell entry and glutathione triggered drug release

To overcome these obstacles, we proposed a cell-penetrating peptide (CPP)-mediated curcumin nanomicelle for the efficient accumulation of chemotherapeutic agents in GBM tumor cells using a combination strategy, for the first time (Fig. 1). Micelles are mainly composed of two functional molecules: (I) Rabies virus glycoprotein polypeptide derivatives (RVG), a CPP, to ensure micelles across the BBB for targeted intracellular drug release. CPPs are reported as effective molecules for delivering exogenous molecules across plasma membrane barriers to target sites. Numerous examples of CPP-mediated cargos entering the brain for various cerebral disorder cures have been reported [17, 18]. However, powerful but nonspecific penetration capability leads to uncontrollability, as a nonnegligible limitation for the application of CPPs in systemic drug delivery and cargo molecule transportation to specific tissues. Polypeptide derived from the neurotropic domain of rabies virus glycoprotein, RVG was introduced as a potential candidate for brain-targeted drug delivery. It has been reported to govern three receptors acetylcholine receptors (NAChRs), and retrograde cargos along motor neurons into the central nervous system for noninvasive delivery [19]. (II) Curcumin with an MDR reversal effect creates the essential hydrophobic core. Extensive investigations suggest the multitargeting capability of curcumin (CUR) on tumor cells and the low toxicity of CUR on normal cells [20, 21], which has also been identified as an inhibitor to suppress MDR-associated proteins, especially P-gp [9, 22]. Additionally, a growing body of evidence indicates that curcumin may play a crucial role in stimulating the immune system to eliminate residual GMB cells [23, 24]. Appropriate delivery of curcumin induces the overall repolarization of microglia, promoting the transformation of GBM cells from an immunosuppressive M2 state to a vulnerable M1 phenotype [25]. Chondroitin sulfate (CHS) was selected as the hydrophilic segment because of its unique microenvironmental compatibility and affinity for intracerebral gliomas [26], conjugating to curcumin via disulfide bonds and spontaneously self-assembling core–shell polymeric micelles in water. RVG-mediated copolymer micelles containing the chemotherapy drug doxorubicin (DOX/RVG-CSC) penetrate the BBB, reach tumor cell regions, and then achieve drug release upon stimulation by the high concentration of glutathione in GBM.

In general, our work mainly covers the synthesis, characterization as well as in vitro and in vivo evaluation of RVG-modified/unmodified DOX-loaded polymeric micelles. Consistent conclusions have been drawn from different studies for superior BBB penetration, MDR reversal and effective brain accumulation of micelles, and even superb inhibitory capability against tumor recurrence after surgery.

## Results and discussion

### Synthesis and characterization of CSC and RVG-CSC micelles

**Fig. 2:**
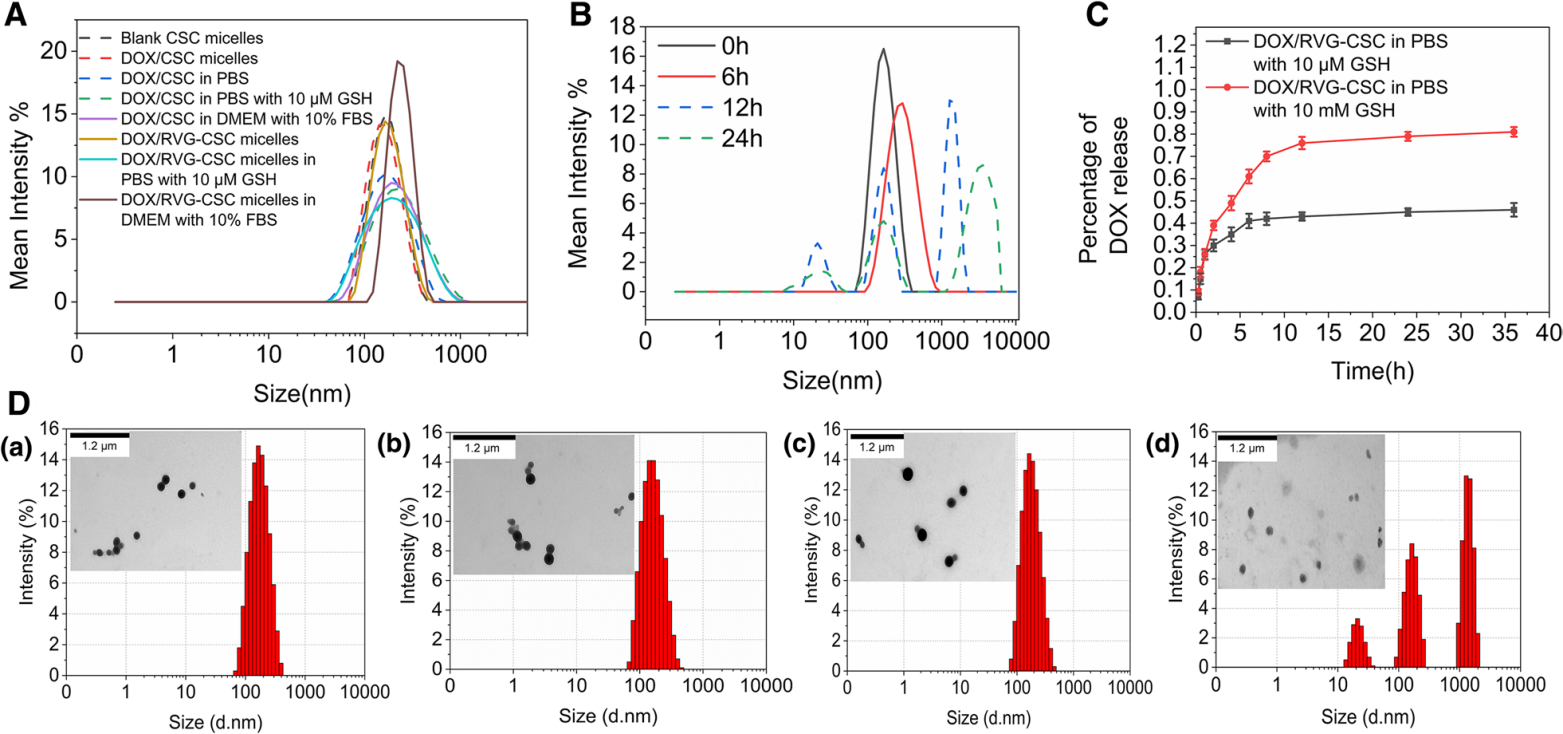
**A** Particle size of DOX/CSC and DOX/RVG-CSC micelles after 24 h in different dissolution media. **B** Changes in size of DOX/RVG-CSC micelles monitored over time at 10 mM GSH in PBS at 37 °C; 0 h was used as a control. **C** In vitro DOX release profile of DOX/RVG-CSC micelles in PBS with 10 μM or 10 mM GSH at 37 °C. **D** TEM image of a) DOX/RVG-CSC, b) DOX/CSC, c) CSC micelles in PBS and d) DOX/CSC micelles treated with 10 mM GSH after 24 h. Scale bar: 1.2 μm

In general, DOX/RVG-CSC micelles were fabricated with a modified sonication method: reduction-sensitive polymers (CHS-ss-CUR) were synthesized, DOX was loaded by self-assembly to form a hydrophobic core, and the micelles were functionalized with RVG. The particle size distribution of nanomicelles is concentrated around 200 nm, as shown in Fig. 2D. The mean particle size of plain and DOX-loaded nanomicelles were 178.93 ± 4.56 and 153.23 ± 2.30 (Table 1), respectively. The size decrease of DOX-loaded nanomicelles may benefit from the following two major reasons: the superior subaqueous dispersibility of chondroitin sulfate [27, 28] and the strong hydrophobic core of micelles. DOX molecules provide the amine group to form a hydrogen bond with the CUR hydroxyl groups and are capable of forming a cationic surface charge to create an electrostatic attraction with the negatively charged CUR [9, 29, 30]. DOX/RVG-CSC micelles were very stable even in a physiological environment containing serum for 24 h (Fig. 2A). Similar to a protein adsorption process, RVG modified on the micellar surface bound to the serum protein through electrostatic interactions, causing an increased micellar size (Table 1, Fig. 2Da). Moreover, DOX/RVG-CSC micelles exhibited a regular spherical and clear core shell structure in PBS (Fig. 2Da). Then the prepared micelles were further observed under a high resolution electron microscope, and the same particle characterization was obtained (Additional file 1: Figure S3). Reduction-sensitive DOX/CSC micelles showed a specific degradation at 10 mM GSH: after 24 h, the hydrophobic core increased significantly to more than 1000 nm (Fig. 2B). Likewise, in morphology, irregular shape and obvious breakage were observed 12 h after treated with 10 mM GSH (Fig. 2B, Dd). Reduction sensitivity was further confirmed by the in vitro release of micelles. After 36 h, the percentage release of DOX from DOX/RVG-CSC micelles at 10 mM GSH was 81%, significantly higher than 46% at 10 μM GSH (Fig. 2C).

**Table 1:** Characterization of CSC, DOX/CSC and DOX/RVG-CSC micelles in PBS

Sample	DOX DL (%)	DOX EE (%)	Diameter (nm)	PDI	Zeta (mV)
CSC	–	–	178.93 ± 4.56	0.204 ± 0.026	− 24.27 ± 1.18
DOX/CSC	8.78 ± 1.73	58.73 ± 12.79	153.23 ± 2.30	0.152 ± 0.016	− 28.15 ± 0.35
DOX/RVG-CSC	8.29 ± 1.50	59.61 ± 10.37	197.87 ± 1.93	0.123 ± 0.022	− 17.87 ± 0.57

### Cytotoxicity and MDR inhibition in vitro

**Fig. 3:**
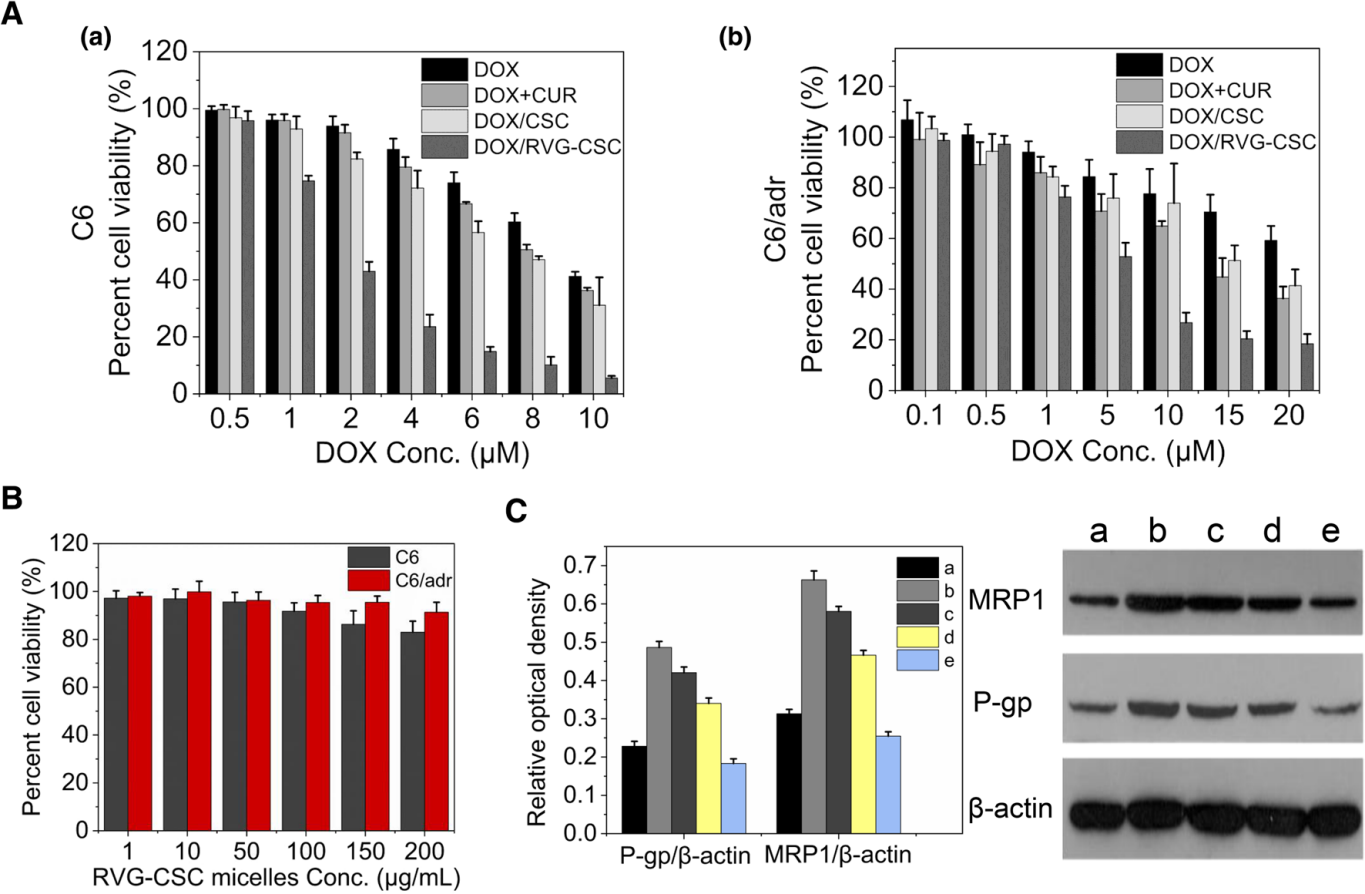
**A** Cell viability of a) C6 and b) C6/adr rat glioblastoma cells in response to treatment with DOX, DOX + CUR, DOX/CSC and DOX/RVG-CSC micelles for 24 h (n = 5). **B** Cell viability of C6 and C6/adr rat glioblastoma cells treated with RVG-CSC micelles (n = 5). **C** Western blot results of semiquantitative analysis and expression of P-gp and MRP1: (a) C6 cells, (b) C6/adr cells, (c) C6/adr cells after DOX treatment, (d) C6/adr cells after CSC micelles treatment, (e) C6/adr cells after RVG-CSC micelle treatment

Prior to the anticancer investigation, MDR activity in C6/adr cells was confirmed with the CCK-8 assay, flow-cytometry, and Western blot. Free DOX did not significantly retard the growth of C6/adr cells at a high concentration of 20 μM (Fig. 3A), and the IC_50_ value for C6/adr was 3.8-fold higher than that for C6 glioma cells (Table 2). And as expected, the expression of MRP1 and P-gp in C6/adr cells increased dramatically, as shown in Fig. 3C.

**Table 2:** IC_50_ values against C6 and C6/adr cells

Samples	IC50 (μg/mL)	
	C6	C6/adr
Free DOX	5.58	21.36
DOX + CUR	4.67	7.23
DOX/CSC	3.71	10.49
DOX/RVG-CSC	1.15	2.51

The in vitro performance of DOX/RVG-CSC micelles was then studied in C6/adr glioma cells. In the absence of DOX, RVG-CSC micelles exhibited no significant growth suppression on C6 and C6/adr cells even at a high concentration of 200 μg/mL, implying low toxicity of these constructed carriers (Fig. 3B). In stark contrast, DOX/RVG-CSC micelles triggered 73.1% death of C6/adr cells at a DOX concentration of 10 μM (Fig. 3A), demonstrating their potent antitumor effect. However, non-CPP-bearing, nonpenetrated DOX/CSC micelles killed only 24.0% of tumor cells, even slightly lower than the effect of DOX + CUR (36.1%), providing good evidence that functionalization with the RVG peptide promotes plasma membrane penetration. This result may be attributable to the cell entry approach of micelles, and DOX/CSC micelles cannot diffuse as fast as free drugs for cell killing. The Western bolt assay provides a further verification. The expression levels of P-gp and MRP1 in C6/adr cells in the group preincubated with DOX/RVG-CSC micelles were obviously downregulated compared with the other groups (Fig. 3D).

### Transportation across the BBB and tumor cell uptake in vitro

**Fig. 4:**
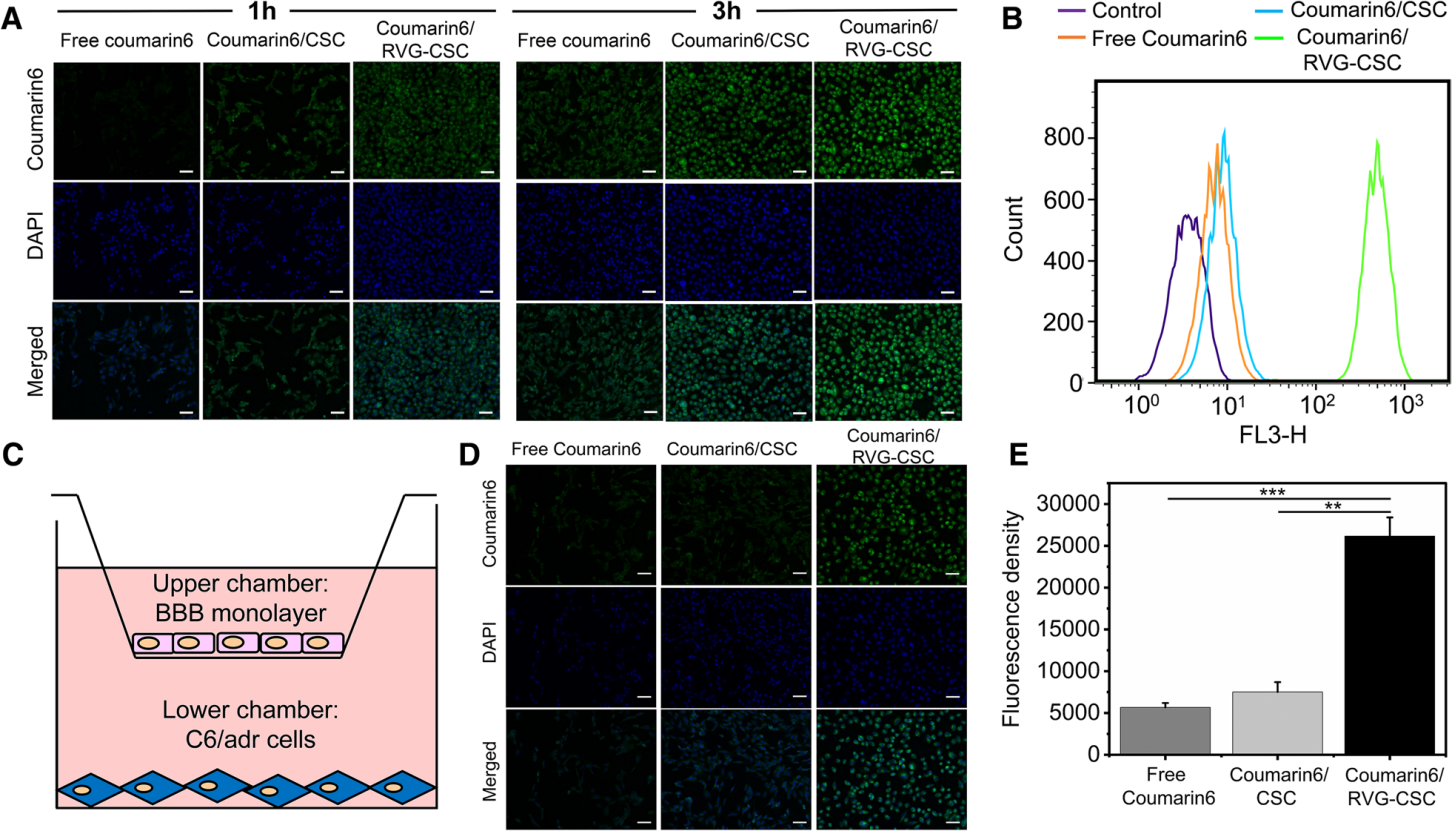
**A** Cell uptake of free coumarin6, coumarin6/CSC or coumarin6/RVG-CSC micelles after incubation for 1 and 3 h. Scale bar: 50 μm. **B** Flow cytometry quantitative analysis of free coumarin6, coumarin6/CSC or coumarin6/RVG-CSC micelles after incubation for 3 h. **C** Schematic diagram of the in vitro BBB model mimicked by bEnd.3 cell monolayer. **D** Fluorescent photographs of free coumarin6, coumarin6/CSC or coumarin6/RVG-CSC micelles in C6/adr cells after penetrating the BBB model. Scale bar: 50 μm. **E** Fluorescence density of coumarin6 in C6/adr cells after penetrating the BBB model (n = 100, *p < 0.05 and **p < 0.01)

To make the results more visible, we encapsulated coumarin6 into micelles to confirm the enhanced BBB penetration and increased intracellular accumulation caused by RVG modification, using flow cytometry and Olympus BX41 inverted fluorescence microscopy for determination. Intracellular fluorescence intensity demonstrated a positive correlation from 1 to 3 h, confirming a time-dependent trend in the cell internalization of RVG-CSC micelles (Fig. 4A). Cellular uptake of RVG-CSC micelles was more efficient than that of non-RVG bearing CSC micelles or in cells incubated with free DOX. After 3 h of incubation with coumarin6/RVG-CSC, intense fluorescence was observed in C6/adr cells, which was distinctly stronger than that in cells treated with coumarin6/CSC or free coumarin6. This result was consistent with flow cytometry results (Fig. 4B). After RVG modification, micellar cell absorption efficiency was markedly improved. The low permeability of the primarily limits the diffusion of therapeutic agents from the bloodstream into the brain for effective accumulation. To evaluate the BBB penetration of micelles, we then constructed a tightly bonded b.End3 cell monolayer in vitro to mimic the BBB, as shown in Fig. 4C. Transporters and tight junction proteins expressed on this bEnd.3 cell monolayer were similar to BBB endothelial primary cells [31]. As shown in Fig. 4D, E, RVG-CSC readily penetrated the BBB, while free coumarin6 was blocked out. Only limited amounts of CSC micelles passed through the BBB monolayer and were effectively taken up by C6/ADR cells in the lower chamber. In contrast, the fluorescence intensity of the RVG-CSC group in the cytoplasm was obviously higher. The results above validate the reliable capability of RVG-modified micelles to undergo efficient cell uptake, rapid intracellular transduction and enhanced BBB penetration.

### Antiproliferative activity and antimetastatic effect in vitro

**Fig. 5:**
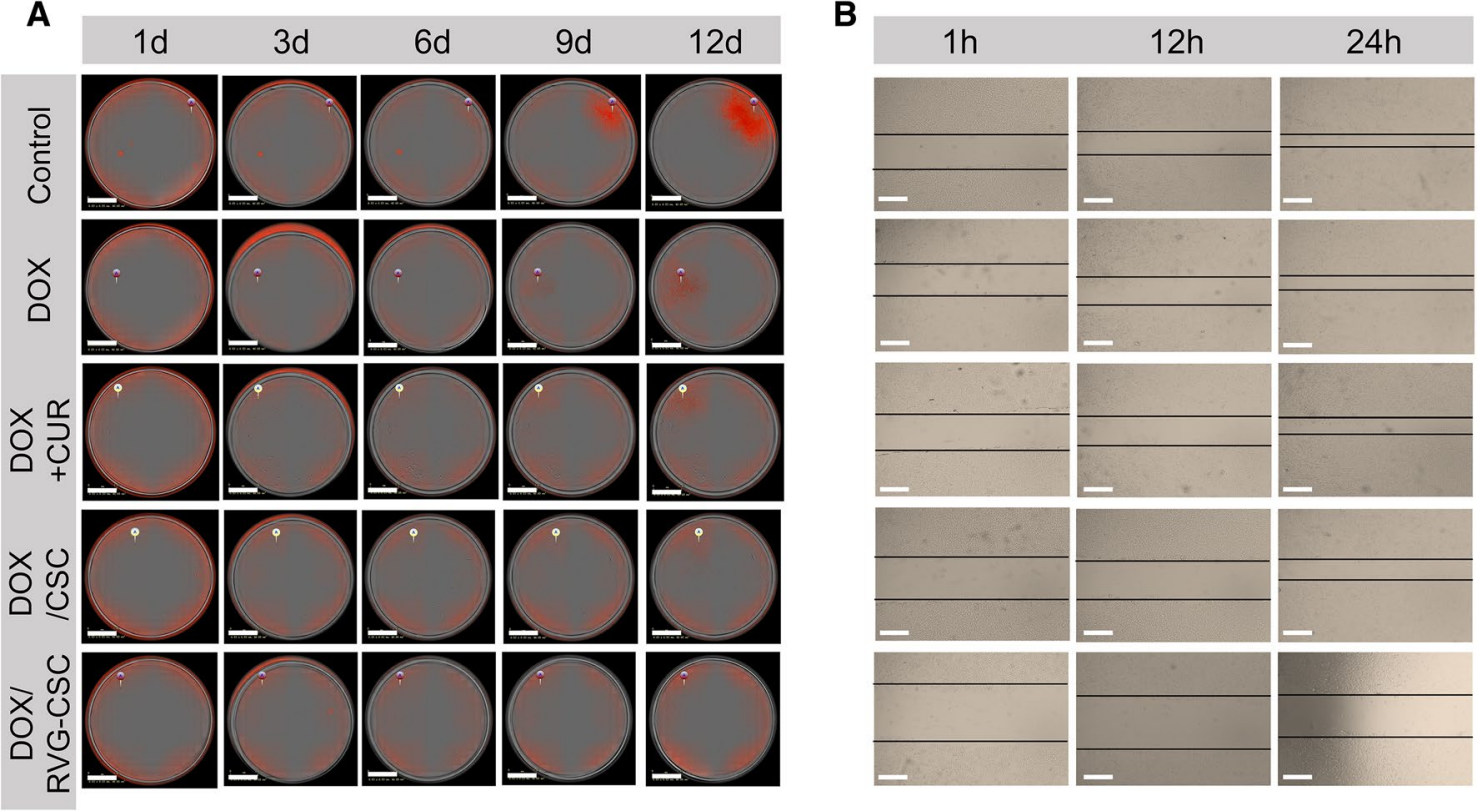
**A** Dilution clone analysis of C6/adr-mkate2 treated with DOX/RVG-CSC, DOX/CSC, DOX + CUR, DOX or PBS. Scale bar = 1.4 mm. **B** Antimigration analysis at 1, 12 and 24 h after treatment with DOX/RVG-CSC, DOX/CSC, DOX + CUR, DOX or PBS. Scale bar = 200 µm

To further elucidate the antitumor activity of DOX/RVG-CSC, we conducted a live/dead cell assay to determine the killing effect of nanomedicines on DOX-resistant rat glioma cells (Additional file 1: Figure S4). DOX/RVG-CSC had significantly higher red fluorescence than the other two treatment groups. Dilution clone analysis validated that DOX/RVG-CSC had a more significant proliferation inhibitory effect (Fig. 5A). The cell counts after 9 days of treatment with DOX/RVG-CSC were 0.16-, 0.13-, 0.07- and 0.04-fold higher than those after DOX/CSC, DOX + CUR, free DOX and PBS treatment, respectively (Additional file 1: Figure S5A). Correspondingly, the antimigration assay showed the same inhibitory trend as the antiproliferative assay (Fig. 5B). As shown in the quantified scratch relative width histogram (Additional file 1: Figure S5B), C6/adr tumor cells incubated with DOX/RVG-CSC after 24 h possessed a maximum relative width (~ 83%) significantly higher than DOX (50.1%), DOX + CUR (65.6%) and DOX/CSC (62.5%). The results above clearly indicated that DOX/RVG-CSC had superior therapeutic efficiency among all treatment groups.

### Biocompatibility and tissue cytotoxicity in vivo

**Fig. 6:**
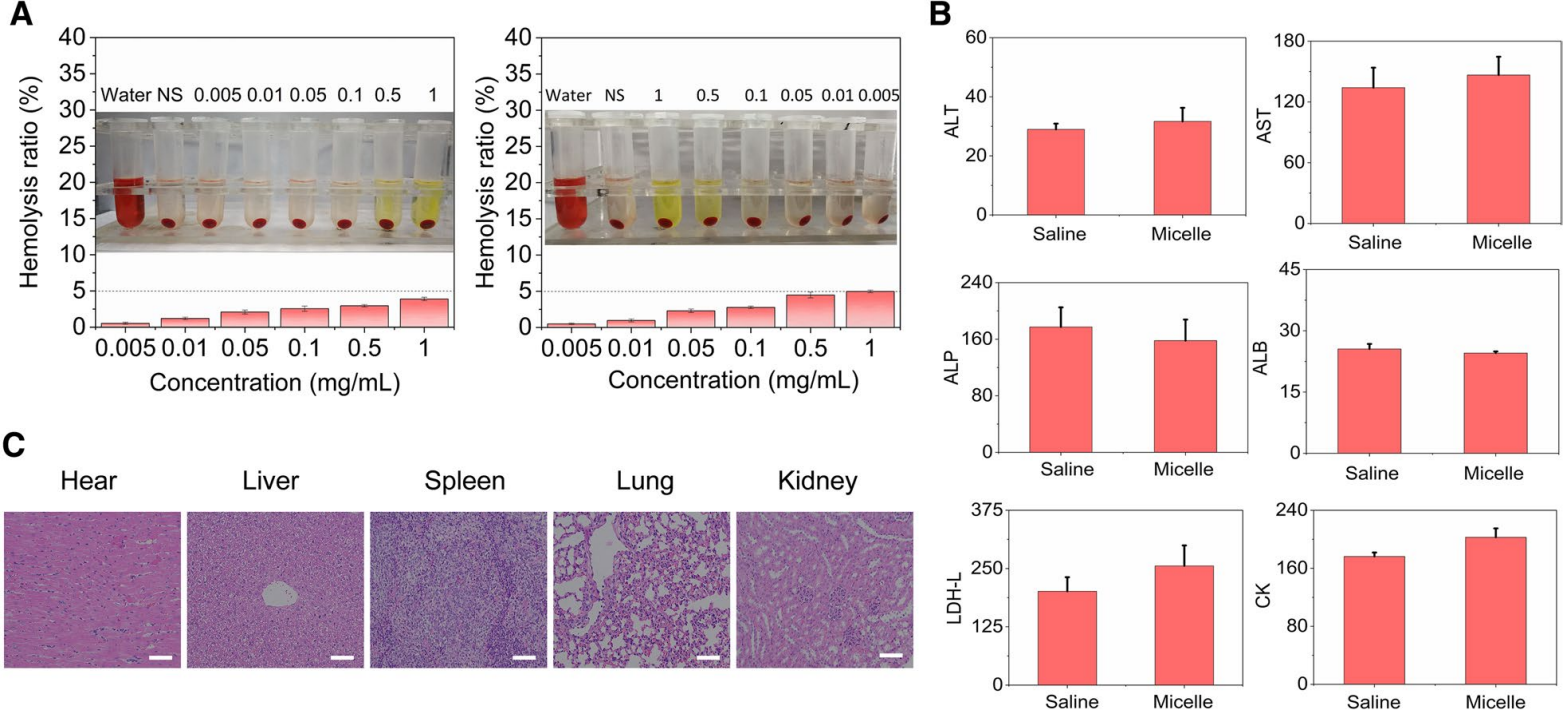
**A** Hemolytic analysis of bare RVG-CSC and CSC micelles. Data are presented as the mean ± SD (n = 3). **B** Blood biochemistry analyses of healthy BALB/c mice after intravenous injection with DOX/RVG-CSC micelles or Saline (10 mg DOX equiv./kg). Data are presented as the mean ± SD (n = 6). Abbreviations for the main biochemical indicators monitored in the blood: *ALT* alanine aminotransferase, *AST* aspartate aminotransferase, *ALP* alkaline phosphatase, *ALB* serum albumin, *LDH-L* lactate dehydrogenase and *CK* creatine kinase. **C** Histological H&E staining analysis of the major organs excised from healthy BALB/c mice treated with bare RVG-CSC micelles. Scale bar: 150 μm

Verification of the constructed nanocarriers in serum stability and tissue nontoxicity was essential before formal in vivo studies. The biocompatibility investigation was performed by coincubating fresh rabbit blood with various concentrations of bare RVG-CSC or CSC micelles. Figure 6A shows that the samples at the concentration range of 0.005–1 mg/mL had superior hemocompatibility and a hemolysis rate below 5% compared with the negative control group. Moreover, there was no significant tissue damage in the major organs excised from health mice after 24 h of treatment with bare RVG-CSC micelles (Fig. 6C). Furthermore, blood biochemical analysis demonstrated that the blood concentration levels of ALT, AST, ALP, ALB, LDH-L and CK were roughly equal in the healthy BALB/c mice treated with DOX/RVG-CSC or saline (Fig. 6B). All the results above indicated the biosafety and clinical potential of nanocarriers for in vivo drug delivery.

### Pharmacokinetic behavior and brain accumulation in vivo

**Fig. 7:**
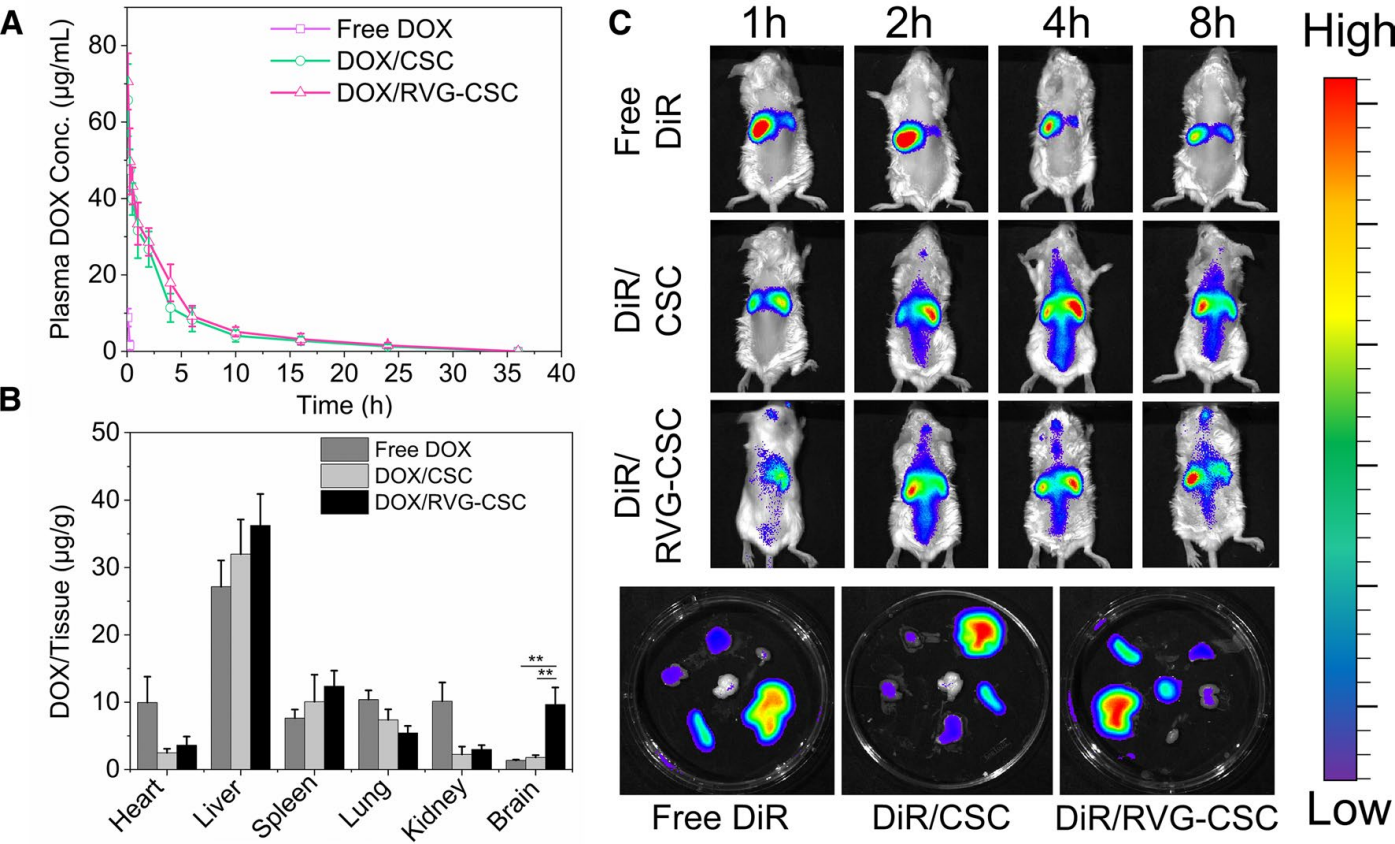
**A** Pharmacokinetic behavior of free DOX, DOX/CSC and DOX/RVG-CSC micelles in BALB/c mice (10 mg DOX equiv./kg). Data are presented as the mean ± SD (n = 3). **B** Quantification of DOX accumulation after 8 h of intravenous injection with free DOX, DOX/CSC and DOX/RVG-CSC micelle biodistribution in different organs excised from BALB/c mice (10 mg DOX equiv./kg, n = 3, and **p < 0.01). **C** Fluorescence images at preset times of living BALB/c mice following treatment with DiR/RVG-CSC, DiR/CSC or free DiR (0.2 mg DiR equiv./kg) via tail vein injection. **D** Fluorescence images of ex vivo organs 8 h after injection with DiR/RVG-CSC, DiR/CSC or free DiR. Heart, liver, spleen, lung and kidney tissues were abbreviated as H, Li, S, Lu and K

To explore the in vivo pharmacokinetic behavior, we measured DOX concentration levels in rat plasma following a single tail vein injection of DOX, taking free DOX as a control. Both DOX/RVG-CSC and DOX/CSC micelles exhibited longer blood circulation times with plasma half-lives of 4.38 and 4.53 h (Fig. 7A), respectively, compared to the rapid systemic clearance of free DOX (unable to obtain meaningful data beyond 0.5 h after i.v.). This increased systemic circulation probably benefited from the physiological properties of the anionic mucopolysaccharide CHS [28], supplying more time for DOX/RVG-CSC micelles to achieve brain transduction. We further investigated the biodistribution and brain accumulation of these novel brain-targeted DOX-loaded micelles by in vivo imaging and tissue residual DOX determination. The near-infrared dye DiR was applied to replace DOX encapsulated in micelles because of its sharper fluorescence and better tissue penetration for easy instrument observation [13]. Visible Dir fluorescence could be observed in the brain after 1 h post injection of DOX/RVG-CSC micelles and was maintained until 8 h to reach the brightest value (Fig. 7C). In comparison, non-RVG-functionalized micelles showed significantly less cerebral accumulation, consistent with the results from 8 h ex vivo organ imaging (Fig. 7D), thereby demonstrating that RVG plays an important role in superior BBB penetration and brain central targets. The biological distribution of nanomedicines in BALB/c mice was quantitatively measured using HPLC and showed that drug accumulation in the brain was up to 8.06 µg/g, 23.03- and 6.55-fold higher than that in the other two treatment groups, respectively (Fig. 7B). Notably, DOX/RVG-CSC exhibited higher accumulation in the liver than free DOX, which might be attributed to the nonspecific penetration of CPP during systemic circulation.

### Antitumor efficacy and postoperative recurrence inhibition in vivo

**Fig. 8:**
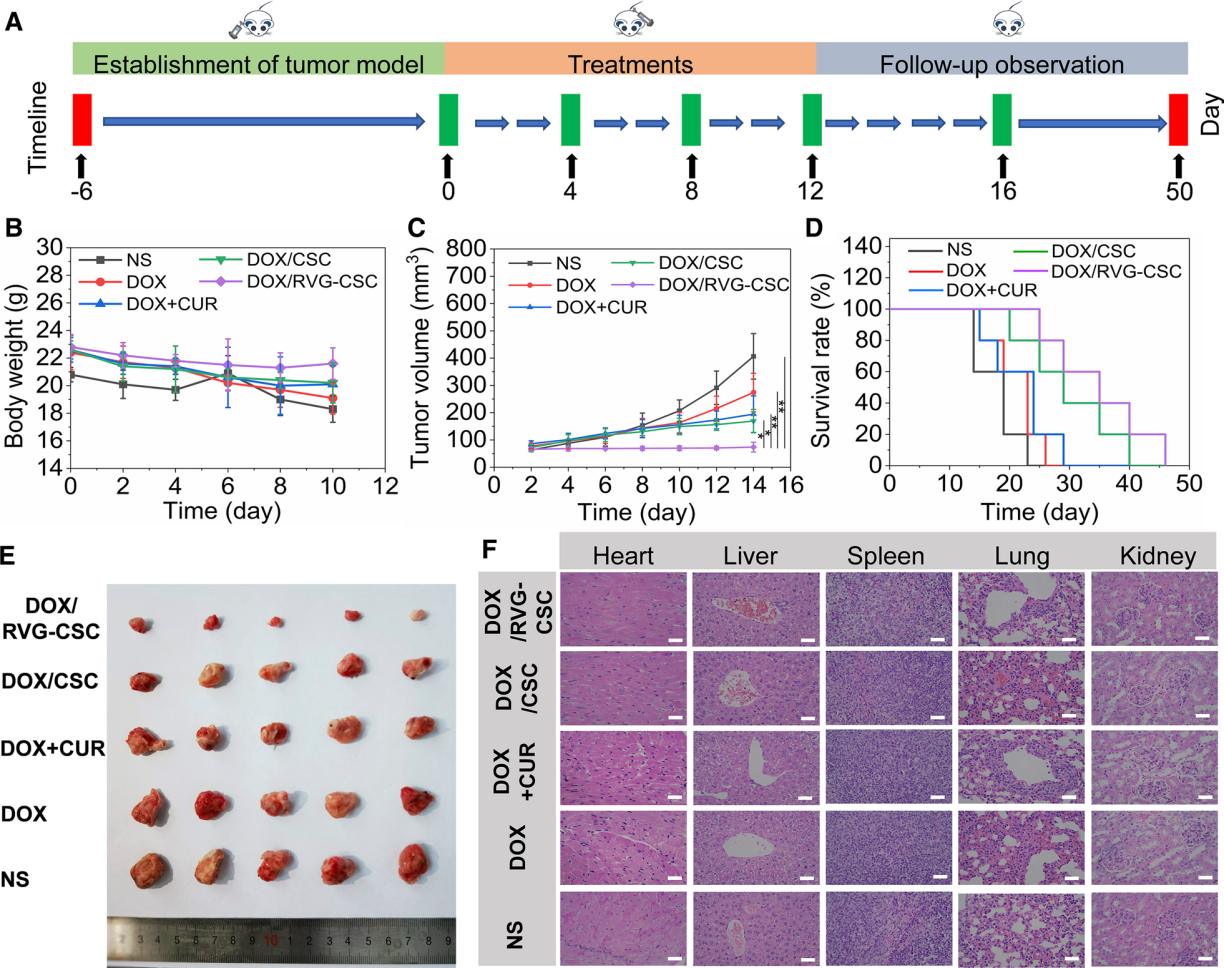
**A** Schematic diagram of the in vitro antitumor experimental process. **B** Body weight changing curves. **C** Quantified tumor size of mice by volume measurement. Data are presented as the mean ± SD (n = 5, *p < 0.01 and **p < 0.05). **D** Mice survival rate curves. **E** Digital photographs of tumor tissues following various treatments. **F** Histological H&E staining analysis of the major organs excised from tumor-bearing BALB/c nude mice following various treatments. Scale bar: 100 μm

The antitumor efficacy of DOX/RVG-CSC micelles was investigated in xenograft-planted C6/adr glioma bearing BALB/c nude mice 6 days post implantation at a dosage of 10 mg DOX equiv./kg via the tail vein every 4 days (Fig. 8A). Groups treated with DOX/RVG-CSC exhibited obvious tumor growth retardation by the smallest glioma size among the five therapeutic groups (DOX/RVG-CSC, DOX/CSC, DOX + CUR, DOX and NS, Fig. 8E) and were quantified by tumor volume measurement every 2 days (Fig. 8C). In contrast, the free DOX and DOX + CUR groups slightly inhibited tumor growth (Fig. 8C), but there was a dramatic decrease in body weight (Fig. 8B), indicating toxicity. Group treatment with NS also showed significant weight loss, reflecting conceivable tissue damage or degradation as the disease progressed [32]. However, little body weight loss was observed in the DOX/RVG-CSC group, demonstrating that the therapeutics clearly retarded tumor growth with negligible adverse effects. Moreover, H&E and TUNEL assay were employed to unveil the antitumor efficacy and apoptosis induction of nanomicelles for glioblastoma cells. The glioblastoma tissue collected from DOX/RVG-CSC showed the severe absence of nuclei and massive tumor necrosis at most regions of H&E section and the highest apoptosis level in the tumor among all the groups (Additional file 1: Figure S6). In addition, the DOX/RVG-CSC group possessed the highest survival rate with a median survival time of 35 days from survival curve analysis (Fig. 8D), which was obviously longer than that of the groups treated with DOX/CSC (29 days), DOX + CUR (24 days), free DOX (23 days) and NS (19 days). Histological H&E staining showed that the main tissues and organs remained normal after injection with DOX/RVG-CSC micelles compared to the significant hepatotoxicity and cardiotoxicity induced by free DOX (Fig. 8F).

**Fig. 9:**
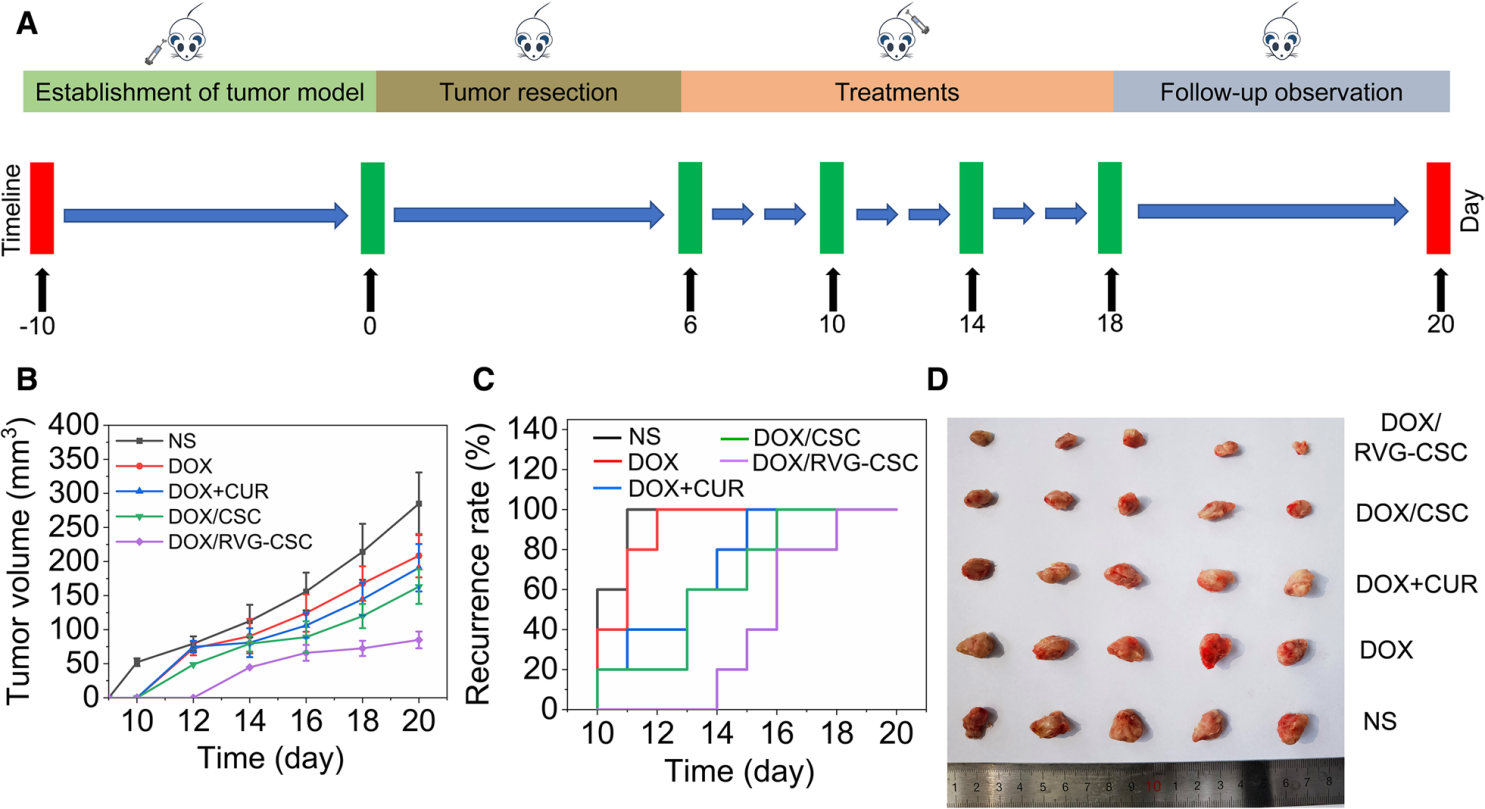
**A** Schematic diagram of the in vitro antirecurrence experimental process. **B** Tumor recurrence rate curves. **C** Recurrent tumor volume curves. **D** Digital photographs of recurrent tumor tissues following various treatments

A subcutaneous C6/adr glioma recurrence assay was then performed to investigate the potential anti-recurrence efficacy of DOX/RVG-CSC (Fig. 9A). Animals were administered every 4 days, and their tumor recurrence together with volume were monitored simultaneously. As shown in Fig. 9B, except for the DOX/RVG-CSC group, all other groups exhibited varied levels of local recurrent tumors at 14 days. Proportionally, the average recurrent tumor volume of the mouse groups treated with DOX/RVG-CSC micelles (84.9 mm^3^) was significantly lower than that of free DOX (208.6 mm^3^), DOX + CUR (190.9 mm^3^) and DOX/CSC groups (163.2 mm^3^); and this result was manifested by visualization photographs of excised recurrent tumors (Fig. 9C, D). To further investigate the inhibition of glioma metastasis, the lung metastasis nodules were examined by Bouin’s solution and H&E staining to after different treatment. As shown in Additional file 1: Figure S7A, B, there was the minimal counts of pulmonary metastatic nodules in DOX/RVG-CSC among all treatment groups. And the results remained largely similar in subsequent H&E staining analysis of lung tissues (Additional file 1: Figure S7C). All results above demonstrated the potential recurrence inhibition and lung metastasis repression of DOX/RVG-CSC micelles against subcutaneous dox-resistant glioma models.

### Inhibition and therapeutic impact for orthotopic brain glioma in vivo

**Fig. 10:**
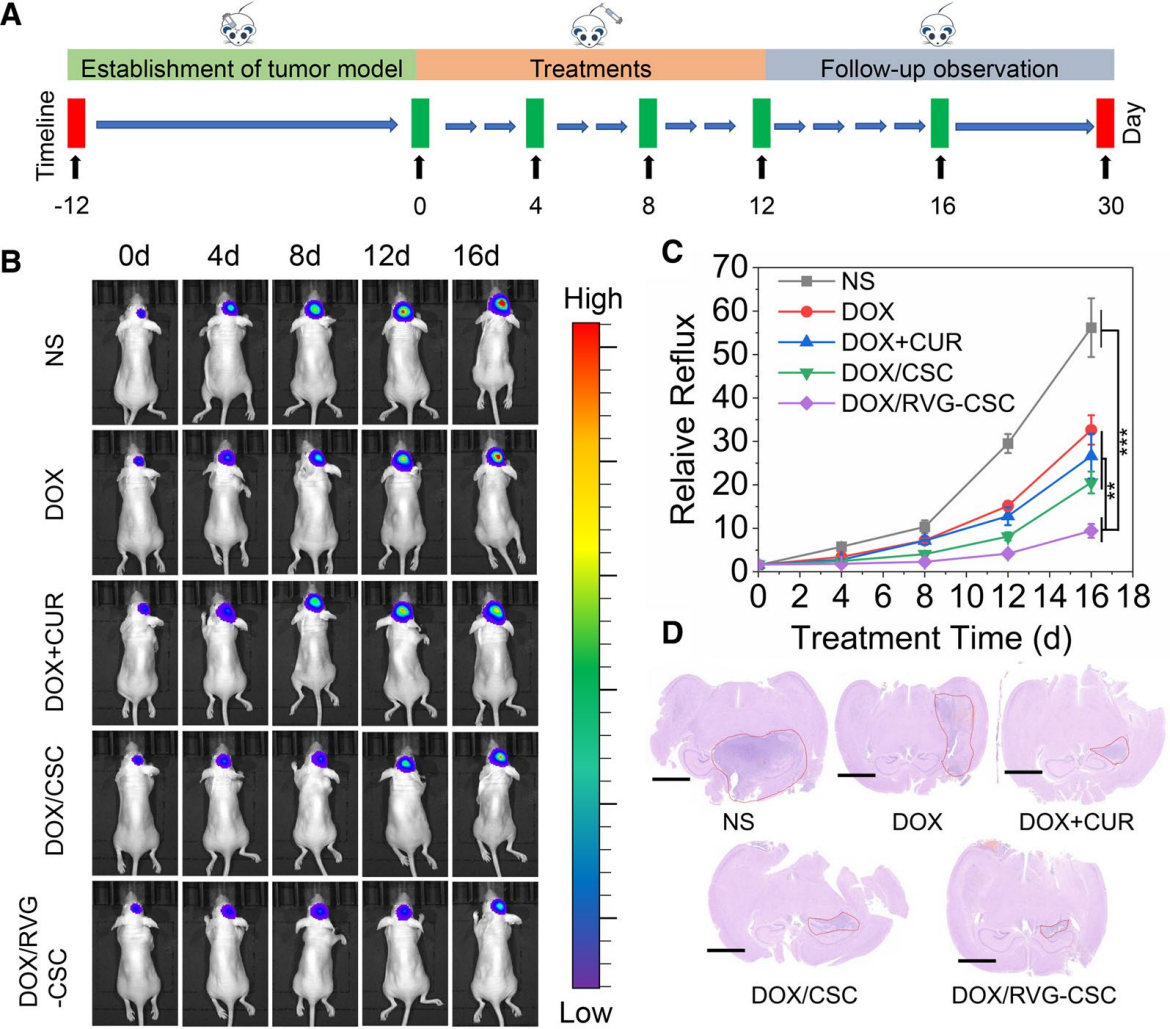
**A** Schematic diagram of the in vitro antitumor experimental process. **B** Luminescence images of BALB/c nude mice bearing orthotopic C6/adr-luc glioblastoma tumor after treatment with NS, DOX, DOX + CUR, DOX/CSC and DOX/RVG-CSC. **C** Quantified luminescence levels of mice using the Lumina IVIS III system. **D** Representative H&E staining images of brain sections excised from mice following various treatment. Scale bar: 1000 μm

We further investigate the efficacy of DOX/RVG-CSC micelles to restrain tumor growth in vivo 12 days post implantation by injecting different micelles formulations (at a dosage of 10 mg DOX equiv./kg) into BALB/c nude mice bearing C6/adr-luc orthotopic glioblastoma via i.v. administration (Fig. 10A). The captured photographs demonstrated that in contrast to rapid increase of the NS group, significantly weaker bioluminescence of glioblastoma was observed in the DOX/RVG-CSC group (Fig. 10B). And unsurprisingly, slight suppression at first but rapid tumor growth was then observed in free DOX, DOX + CUR and DOX/CSC group. The semiquantitative bioluminescence result indicated that obviously C6/adr glioma inhibition was monitored in DOX/RVG-CSC group versus free DOX (Fig. 10C), free DOX + CUR and CSC micelle group, resulting from responsive DOX release, effective blood–brain barrier penetration and enhanced drug accumulation and retention. Meanwhile, steady weight changes (Additional file 1: Figure S8) corroborated that treated with DOX/RVG-CSC micelles effectively restrained glioma growth without causing adverse effects and preserved brain function [33]. Although free DOX and DOX + CUR retarded glioblastoma growth to a certain extent (Fig. 10B, C), the sharp weight loss of mice indicated toxicity (Additional file 1: Figure S8). Moreover, the H&E staining images of mouse brain provided consistent evidence that the smallest glioma volume was observed in mouse brain after DOX/RVG-CSC treatment (Fig. 10D).

## Conclusion

In summary, we have demonstrated that RVG-modified reduction-sensitive nanomicelles loaded with the chemotherapeutic drug DOX enabled enhanced BBB permeability and improved tumor cell uptake and efflux-mediated MDR suppression, leading to more effective antitumor outcomes against glioma. Our versatile brain-targeting drug delivery nanoplatforms emphasize several striking points: (i) DOX/RVG-CSC exhibits superior brain targeting ability and decreased systemic toxicity, compared with free drugs and its nontargeted equivalents; (ii) RVG functionalization facilitates higher brain accumulation via improved BBB permeability; (iii) application of the reduction sensitive polymer enables tumor-triggered drug release for improved selective therapy; and (iv) curcumin conjugation provides MDR inhibition together with antimetastatic effects. This novel and universal nanomicelle can deliver various therapeutic or imaging agents into the brain, which is expected to be an effective strategy for brain-related disorder treatment or diagnosis.

## Materials and methods

### Materials

All reagents and materials without special instructions were purchased from Sigma-Aldrich. Chondroitin sulfate (CHS, Mw 10 kDa) was obtained from the Institute of Biochemistry and Molecular Biology of Shandong University. The rabies virus glycoprotein polypeptide derivative, RVG29 peptide (YTIWMPENPRPGTPCDIFTNSRGKRASNGGGGC) was synthesized by Seebio Biotech Co., Ltd. (Shanghai, China). *N*-Hydroxysuccinimide (NHS), anhydrous triethylamine (TEA, H_2_O ≤ 50 ppm), and *N*-(3-dimethylaminopropyl)-*N*′-ethylcarbodiimide hydrochloride (EDC) were purchased from Aladdin Reagent Co., Ltd. (Shanghai, China). Acetonitrile and methanol (Aladdin Reagent Co., Ltd., Shanghai China) were of high-performance liquid chromatography grade. Trypsin solution (0.25%, w/v) was purchased from Solarbio Science & Technology Co., Ltd. (Beijing, China). Fetal bovine serum (FBS), penicillin streptomycin and Dulbecco’s modified Eagle medium (DMEM) were purchased from Gibco BRL (Gaithersberg, MD, USA). All other chemicals were of analytical grade and used without further purification. Ultrapure water was used in all experiments.

### Cell culture and animal models

Rat brain glioma cell C6 cells and mouse brain microvascular endothelial bEnd.3 cells were supplied by the Institute of Biochemistry and Molecular Biology of Shandong University. C6 and bEnd.3 cells were cultured in DMEM supplemented with 10% FBS and 1% penicillin–streptomycin-amphotericin B mixtures. The cells were cultured as a monolayer in a humidified atmosphere containing 37 °C with 5% CO_2_. All animals were provided by the Laboratory Animal Center of Shandong University.

### Synthesis and characterization of CSC polymers

Generally, polymers were obtained in a simple three-step process (Additional file 1: Figure S1). Initially, CHS (0.324 g) was dissolved in PBS (pH 7.4) to form a 2.28 mg/mL polysaccharide solution, followed by the addition of EDC (1.15 g) and NHS (0.14 g) for carboxyl group activation [34]. And then cystamine (CYS) dihydrochloride was added as a redox-sensitive linker and stirred for 24 h to gain crude reaction mixtures. The purified CHS-CYS product was obtained after dialysis (MWCO 3500) against deionized water and lyophilization. Structure determination was analyzed using ^1^H nuclear magnetic resonance (^1^H-NMR, AV600, BRUKER, Germany). Monocarboxylated curcumin was then prepared according to a published protocol [35]. In detail, curcumin (2 g) and DMAP (0.15 g) were dissolved in 80 mL of THF followed by the addition of anhydrous triethylamine (1.5 mL) after the solution was clarified. Glutaric anhydride (0.7 g) dissolved in 10 mL of THF was added dropwise to the mixed solution, stirred at 90 °C in an oil bath under nitrogen and refluxed for 17 h. The crude product CUR-COOH was then extracted by vacuum rotary evaporation and redissolved in ethyl acetate (100 mL). The solution was repeatedly extracted with 1 M HCl (15 mL) and eluted via silica gel column chromatography for purification (dichloromethane: methanol = 150:1, v/v). 56% yield of CUR-COOH was achieved after purification through column chromatography. Finally, CUR-COOH was chemically grafted to CHS-CYS through another amide formation for the amphiphilic copolymer construct. CUR-COOH (50 mg), EDC (52 mg) and NHS (32 mg) were dissolved in 20 mL of DMSO and stirred at room temperature for 6 h to complete carboxyl activation. CHS-CYS (100 mg) dissolved in PBS (pH 6.5) was then added dropwise to the solution, and the reaction was stirred for 24 h at room temperature under nitrogen protection. The terminal product was obtained after dialysis and lyophilization. The synthesis was confirmed using ^1^H-NMR.

### Preparation and characterization of micelles

Self-assembled CHS-ss-CUR (CSC) micelles were prepared by sonication [36]. Briefly, the synthesized polymer (50 mg) was dispersed in 10 mL (pH 7.4) PBS, and then subjected to sonication (100w, 2 s on/4 s off) for 4 min using a probe sonicator (Yuming Instrument CO., Ltd., Shanghai, China). The resulting micelle solution was filtered with a 0.8 μm microfiltration membrane and stored at 4 °C. DOX-loaded micelles (DOX/CSC) were prepared by an improved dialysis method [37]. DOX/DMSO solution (2 mg/mL) was added dropwise to the polymer solution in PBS (pH 7.4) and stirred to mix thoroughly. After dialysis against water (MWCO = 3500 Da) for 24 h, mixed solution was then subjected to a consistent sonication process above for 2 min. The DOX-loaded micelle solution was obtained by filtration with a 0.8 μm microfiltration membrane and stored at 4 °C. RVG was coated on CSC micelles via electrostatic interactions between the cationic RVG peptide and the negatively charged CSC micelles. RVG peptide dispersed in 100 μL of PBS (pH 7.4) was slowly dropped into 2 mL of CSC solution, and incubated for 15 min under ice bath with stirring. An additional 10 min of incubation was allowed to stabilize RVG-CSC micelles. DOX-loaded RVG-CSC (DOX/RVG-CSC) micelles were prepared using a similar protocol.

Micelles were characterized to verify their specifications prior to experimental usage. Physical characterization included micellar morphology, zeta potential and size under dry and hydrated conditions. To obverse the particle morphology, micelle solution was dropped on the copper mesh and subjected to a consistent incubation phase to ensure gradual staining. Morphological images were obtained via transmission electron microscopy (TEM) using a JEM-2010F (JEOL, Japan) instrument and processed using ImageJ software. Purified micelles were collected as described above and diluted to 1 mg/mL in PBS for subsequent measurements using dynamic light scattering (DLS, performed by Malvern Nano ZSP) to investigate size and zeta potential.

### In vitro release of DOX from bare micelles

To create DOX loading and investigate its release from the micelles, DOX dissolved in DMSO was gradually incorporated into the micelle solution. Incubate and collect the DOX-loaded polymer micelles as described above. The dialysis method was chosen to investigate in vitro release of DOX-loaded micelles under two different reductive conditions: glutathione-containing PBS (20 nM GSH, pH 7.4) and PBS (0 nM GSH, pH 7.4) with 0.2% (w/v) Tween 80. Briefly, 1 mL of micelles was dispersed to the dialysis tube and subjected to a shaker at the constant temperature incubation (37 °C, 100 rpm). Then, 0.5 mL of release medium was withdrawn and replaced by 0.5 mL of fresh media at certain time points. HPLC was applied for DOX concentration determination.

### Drug-loaded micelle stability and environmental sensitivity

The same method was used to collect and purify micelles to determine their stability in response to reductive sensitivity and stability. Micelles from the concentrated stock solution were diluted in a solution of PBS + 0.2% Tween 80 containing either 10 μM or 10 mM GSH. The subsequent micellar solution was incubated on a shaker at 37 °C and 50 rpm. DLS and TEM were used to characterize the changes in the size and morphology of micelles. The same method was used to study the effect of 10% FBS on micellar stability.

### In vitro cytotoxicity assay

A CCK-8 assay was used for the in vitro cytotoxicity evaluation of free drugs and dox-loaded micelles. In detail, C6 and C6/adr cells were seeded in 96-well plates at a density of 5 × 10^3^ for an additional 24 h of culture at 37 °C, 5% CO_2_ and 95% humidity. Then the cell culture medium was removed and supplemented with 100 μL of fresh medium containing a certain concentration of DOX/RVG-CSC micelles. After 24 h of incubation, 20 μL of CCK-8 solution was added to each well and incubated for another 1 h. Blank controls and negative controls were simultaneously implemented, and five replicates were performed for each sample. The absorbance of each well at 430 nm wavelength was measured under microplate reader.

### Evaluation of micellar hemolysis characterization

An in vitro hemolysis assay was constructed to evaluate the blood compatibility of RVG-modified polymer micelles. Freshly collected red blood cells (RBCs) from rats were centrifuged with sterile PBS (3000 rpm, 5 min) three times for thorough cleaning. The cells were then absolutely mixed with phosphate buffer solution to prepare a 2% cell suspension after serum removal. RVG-modified micelles dissolved in 500 μL of saline at different concentrations (0.005, 0.01, 0.05, 0.1, 0.5, 1 mg/mL) were mixed with erythrocyte suspensions of the same volume. After incubation at 37 °C for 2 h, the reaction mixture was centrifuged at 3000 rpm for 10 min. The supernatant was collected and measured at 540 nm using a UV–Vis spectrophotometer. The blood-water and blood-saline samples described above were used as positive or negative controls, respectively.

### Western blot assay

The cellular expression of specific proteins was analyzed by the western bolt assay. C6/adr cells were incubated with fresh medium containing PBS, DOX, DOX + CUR, DOX + verapamil and DOX/RVG-CSC micelles at 37 °C for 48 h. The cells were scraped and lysed in RIPA buffer containing 1% protease inhibitor. The bicinchoninic acid (BCA) method was used for total protein quantification. Samples were subjected to sulfate–polyacrylamide gel electrophoresis (SDS-PAGE) for protein isolation and then transferred onto polyvinyl fluoride (PVDF) membranes. Membranes were then incubated with the primary antibodies in Tris-buffered saline Tween-20 (TBST) with 5% fat-free milk at 4 °C overnight. After washing in clean TBST three times (10 min each), they were coincubated with the secondary antibodies for another 1 h to label the primary antibodies. Ultimately, immune complex visualization was achieved using an enhanced chemiluminescence (ECL) system.

### Intracellular uptake and in vitro BBB model penetration assay

C6/adr cells were seeded in a 12-well plate at a density of 10^5^ cells/well, and coincubated with free coumarin6, coumarin6/CSC, or coumarin6/RVG-CSC (coumarin6 concentration: 1 μg/mL) at 37 °C for 1 or 3 h. Cell nuclei were then stained with DAPI reagent for observation and photographs was captured using an Olympus BX41 inverted fluorescence microscope with a 40× objective. For the quantitative analysis of intracellular accumulation, cells were collected and resuspended in 500 μL PBS for flow cytometry analysis using BD Accuri C6 Plus. Fluorescence histograms were analyzed and recorded using FlowJo software, based on 10,000 gated events.

An in vitro BBB model was generated using a noncontact coculture Transwell system for RVG-modified micelle penetration evaluation. In detail, bEnd.3 cells were seeded at a density of 10^5^ cells/well in the upper chamber coated with 2% gelatin solution. After 7 days of incubation for cell confluence, transepithelial electric resistance (TEER) was measured to reach 300 Ωcm^2^ to ensure the tight junction of the endothelial cell monolayer and barrier formation. C6/adr cells were seeded in the lower chamber at a density of 10^5^ cells/well and incubated overnight. The constructed in vitro BBB model was separately treated with free coumarin 6, coumarin 6/CSC or coumarin6/RVG-CSC micelles for 6 h. The upper chamber was discarded, while the lower chamber was incubated for an additional 6 h and then observed and photographed under a microscope. Images were processed with Image-Pro Plus software and the fluorescence density was recorded for quantitative analysis.

### Monoclonal proliferation assay and live/dead cell analysis

Stable C6/adr-mKate2 cells were isolated by BD FACSAria flow cytometry sorting and seeded in a 96-well plate. The monoclonal formation rate (2–4 cells/well) was determined using the IncuCyte ZOOM Real-Time Live-Cell Imaging System, RT-LCI. Cells were coincubated with different agents for 12 days, and cell proliferation was monitored in real time by a living cell imaging system.

For live/dead cell analysis, different agents were added to C6/adr cells (5 × 10^3^ cells/well) and coincubated for 12 h. Cells were then washed using PBS and stained with calcein-AM and PI according to the manufacturer’s instructions.

### In vivo pharmacokinetics and biodistribution

A Wistar rat model was adopted to evaluate the systemic circulatory metabolism of DOX/RVG-CSC micelles. In detail, twelve rats were randomly divided into three groups (n = 4 in each group) as free DOX, DOX/CSC, and DOX/RVG-CSC. Different preparations were injected via the tail vein following a dosage of 10 mg DOX equiv./kg. Blood (0.3 mL) was then collected from the sinus vein at the predetermined time and immediately transferred into a heparinized tube for 10 min of centrifugation at 3000 rpm/min. The obtained supernatant was analyzed using HPLC for DOX quantification in plasma.

BALB/c mice received an intravenous injection of DOX/RVG-CSC, DOX/CSC or free DOX (10 mg DOX equiv./kg). Animals were sacrificed at 8 h post-injection to collect heart, liver, spleen, lung kidney and brain tissue for biodistribution analysis. To quantify DOX accumulation in different organs, chopped organs were homogenized in 1 mL of cleaning solution (0.3 M HCl: EtOH = 3:7, v/v) using a homogenizer at 10,000 rpm for 15 min. Lysates were shielded from light overnight at − 20 °C, and then centrifuged at 10,000 rpm/min for 10 min. DOX was measured by the same method.

### In vivo/ex vivo imaging

To monitor the biological distribution and brain accumulation of the nanocarriers, dioctadecyl-3,3,3,3-tetramethylindotricarbocyaine iodide (DiR) was encapsulated into the micelles through hydrophobic interactions. BALB /c mice were randomly divided into three groups and injected with 200 μL of different preparations (0.2 mg DiR equiv./kg) via the tail vein. Mice were observed and photographed with Xenogen Ivis Lumina system (Caliper Life Science, USA) at 1,2,4, and 8 h after injection. Isoflurane was delivered to preanesthetize the mice and was continuously inhaled during the image acquisition process.

### In vivo antitumor assay

BALB/c nude mice aged 10 weeks (n = 10) were subcutaneously inoculated with C6/adr cells to establish the xenograft glioma model. When the tumor volume reached 50–100 mm^3^, the mice were randomly divided into five groups treated with DOX/RVG-CSC, DOX/CSC, DOX + CUR, DOX and NS (10 mg DOX equiv./kg) via the tail vein. Treatment was performed every 4 days for a total of three times while body weight together with tumor volume were monitored every 2 days. Tumor volume was then estimated according to the following equation: volume = 1/2 × (length × width^2^). At the end of treatment, 5 mice per group were sacrificed to collect major organs and tumors for photographs and H&E staining. The survival time of the remaining animals was recorded for Kaplan Meier survival curve.

### Anti-recurrence efficacy in vivo

C6/ADR cells were transplanted into the left armpit of mice to generate subcutaneous tumors. After 10 days later, when tumor volume reached 100–200 mm^3^, tumors were surgically removed leaving 5% residual tumor tissue. Mice were then randomly divided into 5 groups and subjected to treatment with DOX/RVG-CSC, DOX/CSC, DOX + CUR, DOX and NS (10 mg DOX equiv./kg) via the tail vein every 4 days three times 6 days post surgery. The time at which palpable subcutaneous nodules could be detected was recorded and tumor volumes were measured every 2 days thereafter. All mice were sacrificed at day 20 to obtain digital images of recurrent tumors for observation and comparison.

### Statistical analysis

Data are presented as the mean and standard deviation (mean ± SD). Statistical data comparison among groups was verified with 1-way ANOVA followed by Tukey’s multiple comparison test using SPSS Statistics 26. Statistical significance represented by *p < 0.05, **p < 0.01 and ***p < 0.005 is indicated in the figures.

## Supplementary Information

**Additional file 1: Figure S1.** Schematic diagram of the synthetic route of CSC polymer. **Figure S2.** ^1^H-NMR spectrum of CUR-COOH, CHS-CYS and CHS-ss-CUR. **Figure S3.** High-resoluton TEM structure of different nanomicelles. **Figure S4.** Live/dead staining assay of C6/adr cells after 12 h treated with different agents. Scale bar: 100 μm. **Figure S5.** (A) Cell growth and proliferation curve, automatic counting every 4 h within 9 days using an IncuCyte ZOOM real-time live-cell imaging system (RT-LCI). Data were presented as the mean ± SD (n = 3, ***p < 0.005). (B) Histogram of scratch relative width. Data were presented as the mean ± SD (n = 3). **Figure S6.** (A) Representative H&E stained sections of tumor tissues after different treatment. (B) TUNEL analysis of tumor tissues following various treatments. Scale bar: 100 μm. **Figure S7.** (A) Representative photograph of the tumor macro-metastatic nodules in the lungs collecting from BALB/c mice following various treatments. (B) Quantitative analysis of pulmonary metastasis nodules following various treatments. (C) Representative H&E staining images of lung sections excised from mice following various treatment. Scale bar: 400 μm. **Figure S8.** Body weight changing curves of orthotopic glioma-burdened BALB/c following various treatment.

## Data Availability

All data generated or analyzed during this study are included in this published article and its additional files.
