# Supplementary material for: RVG-functionalized reduction sensitive micelles for the effective accumulation of doxorubicin in brain
Source: J Nanobiotechnology. 2021 Aug 21;19:251. doi: 10.1186/s12951-021-00997-z (PMC8379803; doi:10.1186/s12951-021-00997-z)
Supplement: Supplementary file 1 — Additional file 1: Figure S1. Schematic diagram of the synthetic route of CSC polymer. Figure S2. 1H-NMR spectrum of CUR-COOH, CHS-CYS and CHS-ss-CUR. Figure S3. High-resoluton TEM structure of different nanomicelles. Figure S4. Live/dead staining assay of C6/adr cells after 12 h treated with different agents. Scale bar: 100 μm. Figure S5. (A) Cell growth and proliferation curve, automatic counting every 4 h within 9 days using an IncuCyte ZOOM real-time live-cell imaging system (RT-LCI). Data were presented as the mean ± SD (n = 3, ***p < 0.005). (B) Histogram of scratch relative width. Data were presented as the mean ± SD (n = 3). Figure S6. (A) Representative H&E stained sections of tumor tissues after different treatment. (B) TUNEL analysis of tumor tissues following various treatments. Scale bar: 100 μm. Figure S7. (A) Representative photograph of the tumor macro-metastatic nodules in the lungs collecting from BALB/c mice following various treatments. (B) Quantitative analysis of pulmonary metastasis nodules following various treatments. (C) Representative H&E staining images of lung sections excised from mice following various treatment. Scale bar: 400 μm. Figure S8. Body weight changing curves of orthotopic glioma-burdened BALB/c following various treatment. [file 12951_2021_997_MOESM1_ESM.docx]

Curves and table Section


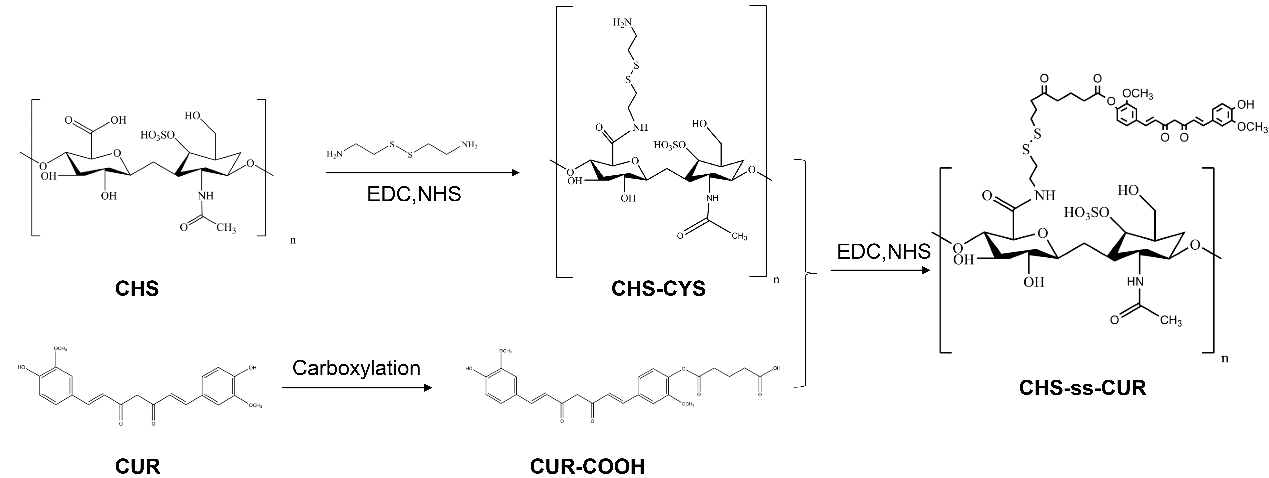


**Figure S1.** Schematic diagram of the synthetic route of CSC polymer


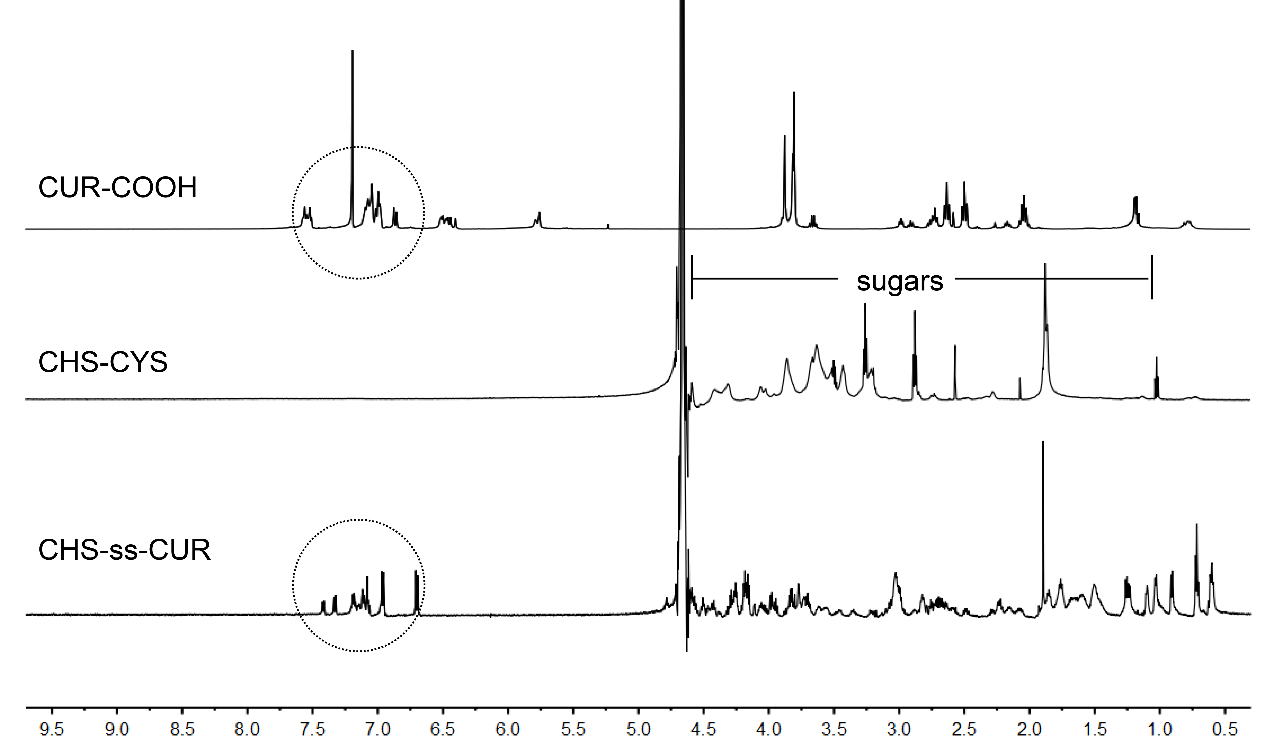


**Figure S2.** ^1^H-NMR spectrum of CUR-COOH, CHS-CYS and CHS-ss-CUR.


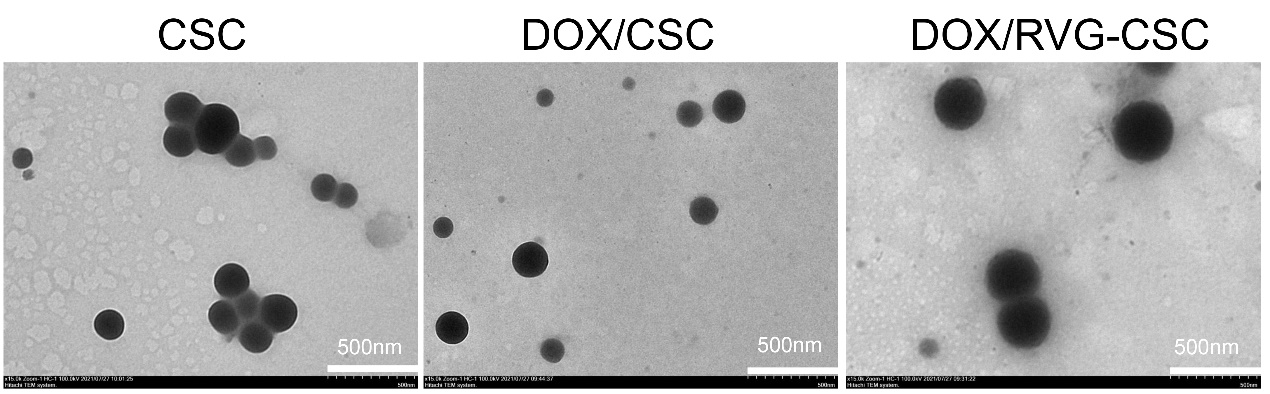


**Figure S3.** High-resoluton TEM structure of different nanomicelles.


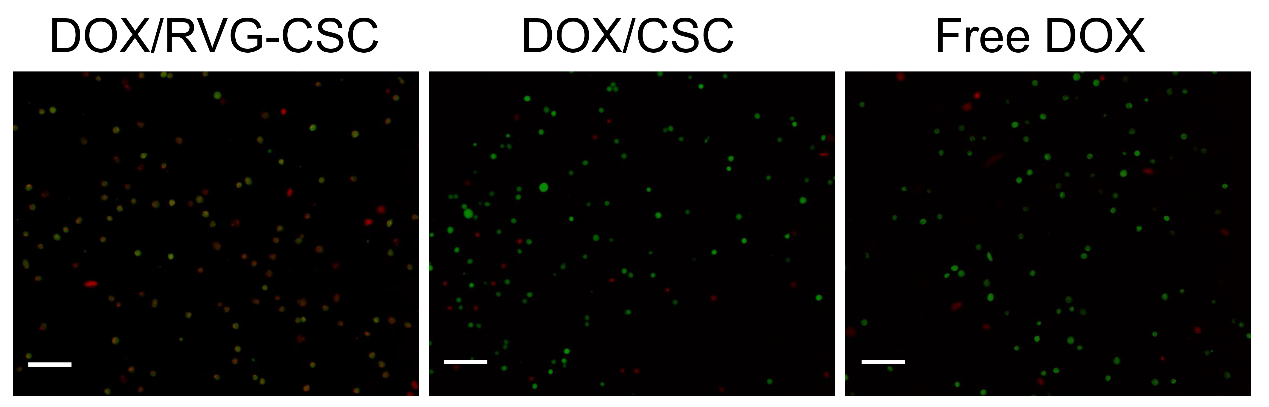


**Figure S4.** Live/dead staining assay of C6/adr cells after 12 h treated with different agents. Scale bar: 100 μm.


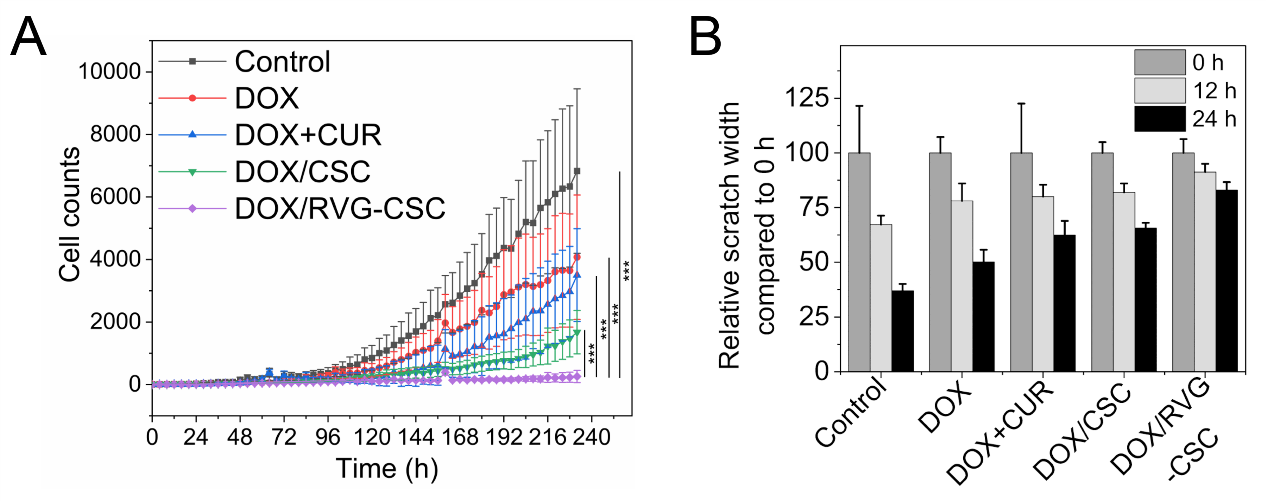


**Figure S5.** (A) Cell growth and proliferation curve, automatic counting every 4 h within 9 days using an IncuCyte ZOOM real-time live-cell imaging system (RT-LCI). Data were presented as the mean ± SD (n=3, ***p<0.005). (B) Histogram of scratch relative width. Data were presented as the mean ± SD (n=3).


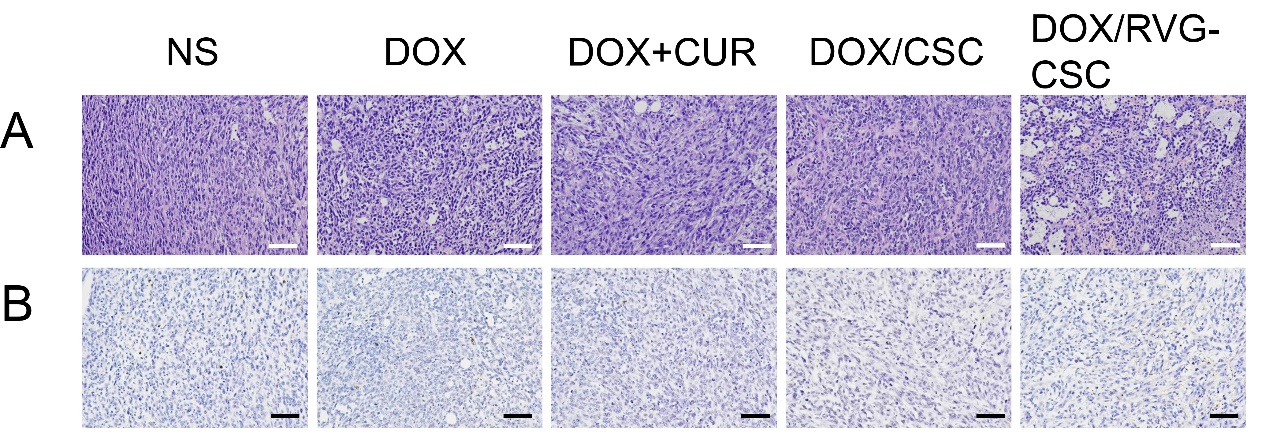


**Figure S6.** (A) Representative H&E stained sections of tumor tissues after different treatment. (B) TUNEL analysis of tumor tissues following various treatments. Scale bar: 100 μm.


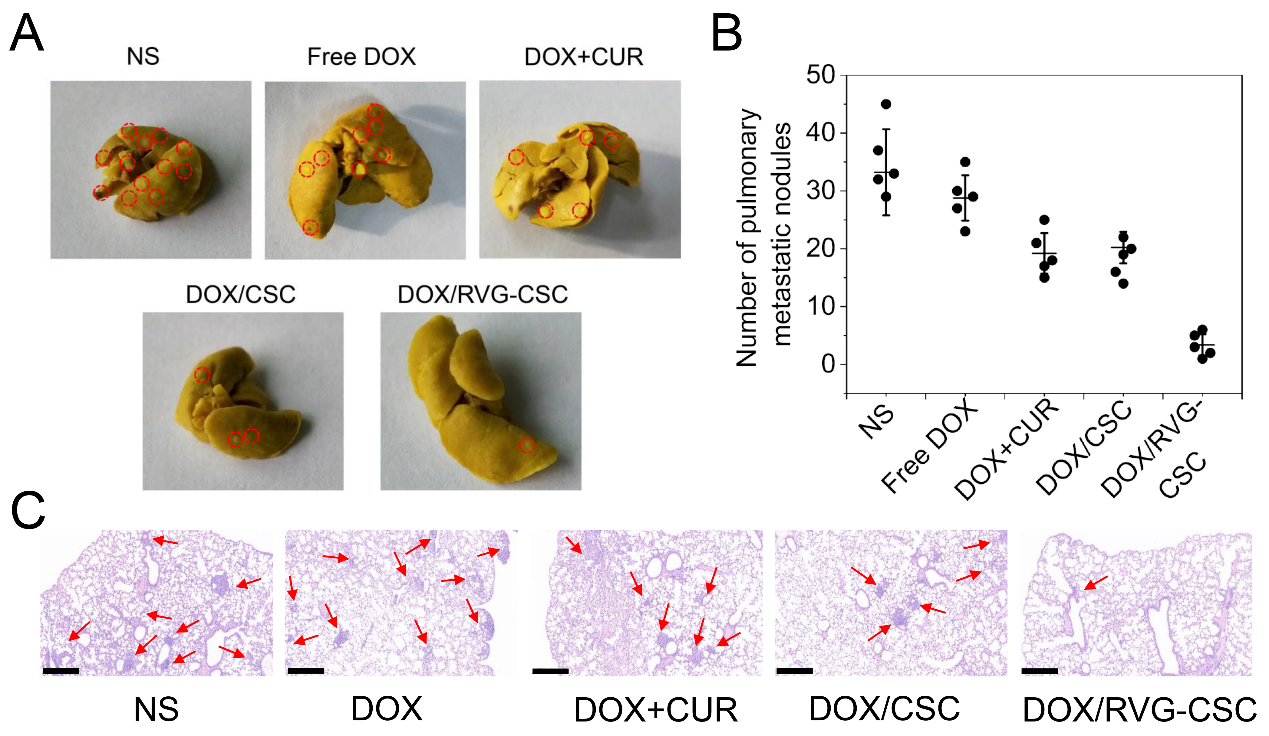


**Figure S7.** (A) Representative photograph of the tumor macro-metastatic nodules in the lungs collecting from BALB/c mice following various treatments. (B) Quantitative analysis of pulmonary metastasis nodules following various treatments. (C) Representative H&E staining images of lung sections excised from mice following various treatment. Scale bar: 400 μm.





**Figure S8.** (B) Body weight changing curves of orthotopic glioma-burdened BALB/c following various treatment.
